# Characterization of lesion formation in marmosets following inhalational challenge with different strains of *Burkholderia pseudomallei*

**DOI:** 10.1111/iep.12161

**Published:** 2016-01-19

**Authors:** Michelle Nelson, Alejandro Nunez, Sarah A. Ngugi, Adam Sinclair, Timothy P. Atkins

**Affiliations:** ^1^ Microbiology GroupCBR DivisionDstlSalisburyWiltshireUK; ^2^ Animal Plant and Health AgencyAddlestoneSurreyUK

**Keywords:** animal model, melioidosis, NHP

## Abstract

The marmoset model of melioidosis was used to explore whether there was any difference in the disease presentation and/or the lesion formation following inhalational challenge with one of four strains of *Burkholderia pseudomallei* (K96243, 1026b, HBPUB10303a and HBPUB10134a). Marmosets were challenged with a range of bacterial doses and bacterial load, histological and physiological features were determined temporally following lethal disease. Melioidosis presented as an acute, febrile disease with bacteraemia, bacterial dissemination, necrotizing hepatitis, splenitis and pneumonia which was independent of the challenge strain. Generally, there were no major differences in the manifestation of melioidosis following challenge by the different strains of *B. pseudomallei*; however, there were some differences in the time to death and the severity of the pathological features. The pathological features observed in the liver and spleen of animals challenged with *B. pseudomallei* strain 1026b were statistically less severe (*P* < 0.05) and less frequent. However, more severe foci of disease were evident in the lungs of animals challenged with strain 1026b. In all cases, the lesions developed from small areas of bacteria‐infected macrophages surrounded by non‐infected neutrophils into large lesions with both immune cell types infected. The marmoset model was a useful tool enabling the distinction of subtle difference in the pathological response to *B. pseudomallei*.

## Introduction

Melioidosis is an important public health concern in many countries including South‐East Asia and in northern Australia. This increasingly prevalent disease is caused by the Gram‐negative facultative intracellular bacterium, *Burkholderia pseudomallei*, and has been classified as a HHS/CDC Tier 1 agent. Melioidosis presents with diverse clinical manifestations, varying from acute sepsis, through chronic localized pathological features, to latent infection. The form of the disease is dependent on a number of different factors including route of infection, bacterial dose received and human predisposing factors. Dogma also suggests that the disease presentation is related to the infecting strain of *B. pseudomallei* (Dance 1996).

A number of animal models have been used to understand the pathogenesis of *B. pseudomallei*, typically mice and hamsters (Fritz *et al*. 1999; Jeddeloh *et al*. 2003; Lever *et al*. 2009). However, as not all strains have been assessed in a single animal model, direct comparison of the strain effect is difficult. The importance of using characterized strains of bacteria to allow standardization of the infectious material and comparison of the data has been highlighted (Van Zandt *et al*. 2012). The use of standardized strains was used to investigate the pathogenesis of *B. pseudomallei* strains K96243 and HBPUB10303a in Balb/c mice (Massey *et al*. 2014). Generally, the disease presentation was similar with the two Burkholderia strains, with the disease onset being quicker following challenge with *B. pseudomallei* strain HBPUB10303a.

The marmoset model (*Callithrix jacchus*) was employed to investigate whether the strain has an effect on the pathological disease features. Non‐human primates generally have a high homology with humans, specifically immunologically and physiologically (Herodin *et al*. 2005). The marmoset is a New World Monkey (NWM) species that is being used as an alternative NHP model to complement the more traditionally used Old World Monkeys (OWM) (e.g. rhesus and cynomolgus macaques). The marmosets is a lower‐order primate; however, the degree of immunological similarity between humans and marmosets is still high (‘t Hart *et al*. 2004). This homology manifests both at genetic and at protein levels (Uccelli *et al*. 1997; Villinger *et al*. 2001; von Budingen *et al*. 2001). As marmosets are a small non‐human primate, they have the advantage of being easier and safer to handle at high containment than larger OWM, but have a high immunologically and physiologically similarity to humans.

Melioidosis has previously been investigated in marmosets using *B. pseudomallei* strain 13392 [originally thought to be strain K96243 (Nelson *et al*. 2013)]. Melioidosis presented as a rapid, acute disease (Nelson *et al*. 2011, 2014). This model was further exploited in this study in order to compare the virulence and lesion characteristics in lethal disease caused by one of four strains of *B. pseudomallei* (HBPUB10303a, HBPUB10134a, 1026b and K96243). Additionally, the temporal formation of lesions following infection with either *B. pseudomallei* strain HBPUB103030a or strain K96243 was investigated.

## Methods and materials

### Bacterial strain and culture

Glycerol stocks of all *B. pseudomallei* strains (K96243, 1026b, HBPUB10303a and HBPUB10134a) were supplied by Biomedical Research and Development Authority (BARDA) from Battelle Biomedical Research Center or Public Health England, UK. These strains are two widely used laboratory strains, K96243 and 1026b, and two recent clinical isolates, HBPUB10303a and HBPUB10134a). Bacteria were recovered on to Luria–Bertani medium supplemented with 4% glycerol (LBG) agar plates and incubated at 37°C for 24 ± 2 h. The optical density (OD_600_) of a loopful of colonies in phosphate‐buffered saline (PBS) was determined (CO7500 Colorimeter, WPA Colourwave Biochrom Ltd, Cambridge, England), and the OD_600_ was adjusted to between 0.35 and 0.38, which is equivalent to approximately 1 × 10^8^ cfu/ml. One millilitre of final suspension was used to inoculate 100 ml LBG broth which was incubated at 37°C on a rotary shaker (Innova 4200 Shaking Incubator; New Brunswick Scientific Stevenage, UK) at 0.4 g for 16 ± 2 h. The OD_600_ of the culture was adjusted with PBS, and a series of 10‐fold dilutions (10^−1^ to 10^−8^) were prepared. The appropriate dilution was selected to the load into the Collison nebulizer (Dstl Engineering, Salisbury, England) for challenge and bacteria were enumerated retrospectively.

### Animals

Healthy, sexually mature, naive common marmosets (*C. jacchus*) were obtained from the Dstl Porton Down breeding colony and housed in vasectomized male and female pairs. For these studies, animals were aged between 1.7 and 5.3 years (mean of 2.7 ± 0.1 years) and weighed between 310 and 525 g (mean of 398 ± 6 g) at the time of challenge. Animals were allocated to studies by animal care technicians not involved in the study design based on availability to avoid bias. All animals were surgically implanted intraperitoneally with a Remo 200 temperature device under general anaesthesia which provides continuous real‐time core body temperature. For surgery, animals were removed from their home cage between 8 am and 1 pm on the day of challenge and initially sedated with 3 mg of ketamine hydrochloride and 30 μg of medetomidine hydrochloride by the intramuscular route and maintained under isoflurane. Prophylactic pain relief (0.2 mg/kg meloxicam and 0.005 mg/kg buprenorphine) was administered by the subcutaneous route. Postsurgery, animals were administered oral meloxicam for 5 days. This regimen of anaesthetics and analgesia has previously shown to be effective to alleviate pain for clinical surgeries performed within Dstl's breeding colony. Animals were returned to a postsurgery recovery cage, had their body temperature maintained with a heat lamp and were observed until the animals were fully mobile (up to 30 min). Once the animals exhibited no adverse effects from the anaesthesia (up to 4 h), they were returned to their home cage. For aerosol exposure, animals were removed from their home cage between 9 am and 2 pm on the day of challenge and sedated with 10 mg of ketamine hydrochloride by the intramuscular route. Following challenge with *B. pseudomallei*, all animals were handled under animal containment level 3 (CL3) conditions, within a half‐suit isolator compliant with British Standard BS5726. Diet and a variety of food (e.g. boiled eggs, fresh fruit and vegetables) were provided daily, and irradiated water was provided *ad libitum*. The type of environmental enrichment was varied and included at least one of the following: ropes, wooden perches, cartons containing forage, nesting boxes, plastic buckets and forage trays. Animal rooms were lit with fluorescent lights and maintained on a 12‐h light/dark cycle. Temperature and RH values were ranged from 20.0–22.8°C and 43.2–68.5% humidity respectively.

Animals were humanely killed upon reaching the humane endpoint (dose ranging studies) or at predetermined times postchallenge (12, 24, 36 and 48 h in the temporal studies). This was performed using an overdose of sodium pentobarbitone after they were sedated with 10 mg of ketamine hydrochloride. The humane endpoint was reached when the animals exhibited at least a 2°C decline compared to their normal baseline core body temperature at that specific time of the day or night, following a fever and the presence of at least one clinical sign (piloerection, hunched posture, respiratory problems, changed locomotion).

### Ethical approval

This work was performed under a UK Home Office Project Licence, which had been reviewed by the Dstl Animal Welfare and Ethical Review Body before submission to the UK Home Office and Animal Procedures Committee (an independent committee that offers advice to The Secretary of State of the ethics of the proposed work).

### Aerosol exposure

Marmosets were exposed to the target dose of *B. pseudomallei* by the inhalational route using a contained Henderson apparatus controlled by the AeroMP (Aerosol Management Platform) aerosol system (Biaera Technologies L.L.C. Hagerstown, MD, United States). Animals were sedated and placed within a plethysmography tube and attached to the exposure unit as previously described (Nelson *et al*. 2009). The animals were exposed to the aerosolized bacteria for 10 min and the aerosol cloud sampled at the midway point of challenge into PBS via an All‐Glass Impinger (AGI‐30; Ace Glass, Vineland, NJ, USA). The impinger concentration was enumerated by serial dilution and plating onto LBG agar as described above. The accumulated volume of air breathed by each animal was determined real time using edacq software (version 1.8.4b EMMS, Bordon, England). The dose each animal received was calculated as follows:

$$\text{Aerosol concentration}\;\left(\text{cfu}/l\;\text{of air}\right)=\frac{\text{Impinger count}\;(\text{cfu}/\text{ml})\;\times \;\text{Impinger volume}\;(\text{ml})}{\text{Impinger flow rate}\;(l/\text{min})\;\times \;\text{Impinger time}\;(\text{min})}$$

$$\begin{array}{cc} \text{Dose received}\;(\text{cfu}) & =\text{Aerosol concentration}\;(\text{cfu}/l\;\text{of air})\\ & \;\times \;\text{total accumulated volume}\;(l) \end{array}$$

### Postmortem analysis

Postmortem examinations were performed on all animals in all studies; organs removed were assessed for bacterial burden, gross and histopathological features and immunohistochemical staining. Blood was removed from anaesthetized marmosets by cardiac puncture for the assessment of bacteraemia, clinical chemistry and haematological parameters and compared to prechallenge data.

### Bacteriology

Bacterial burdens were determined in blood, liver, spleen, kidneys, lungs and brain. Organs were removed aseptically and processed as previously described (Nelson *et al*. 2009). Appropriate dilutions were subcultured onto LBG agar plates in duplicate for *B. pseudomallei* and incubated at 37°C for 24 h respectively. Counts were expressed as cfu/g of tissue or cfu/ml of blood.

### Histopathology and immunohistochemistry

Tissues were fixed in 10% neutral buffered formalin and processed for paraffin wax embedding using standard techniques. Thin sections (4 μm) were cut and stained with haematoxylin and eosin for histopathological analysis. Immunohistochemistry (IHC) was performed as previously described (Nelson *et al*. 2014) on selected tissues (liver, spleen, lungs and the inoculation site) for the detection of bacterial antigen, T cells (CD3^+^), B cells (CD79^+^), macrophages and neutrophils (NEUs) (MAC387^+^) and inducible nitric oxide synthase (iNOS^+^). A semiquantitative scoring system was established to allow comparison (Table 1).

**Table 1:** The Scoring System used for pathological interpretation

Numerical value	Symbol	Histopathological description	Immunohistochemistry description
1	+	Small focal microgranuloma composed mainly of macrophages, lymphocytes and plasma cells	Very few (<5) stained cells
2	++	Non‐necrotic multifocal solid lesions	Small number (5 – 20) stained cells
3	+++	Focal or multifocal suppurative lesions with the presence of abundant neutrophils and small areas of necrosis	Moderate number (20–50) stained cells
4	++++	Multifocal lesion with severe necrosis	Abundant (>50) stained cells

### Haematology, clinical chemistry, electrolytes and coagulation

Blood was collected from all animals at postmortem into blood tubes anti‐coagulated with either EDTA, lithium heparin or sodium citrate. Haematological parameters were measured by a laser‐flow cytometry‐based haematological analyser using EDTA‐coagulated blood (ProCyte, IDEXX Laboratories Ltd, Bucks, UK). Heparinized blood was used to assess the clinical chemistry, and electrolyte parameters were analysed using a ‘dry‐slide’ technology biochemistry analyser (Catalyst Dx; IDEXX Laboratories Ltd). Citrated blood was used to measure the clotting times using the Coag DX (Idexx, Laboratories Ltd).

### Statistics

Haematological and clinical chemistry parameters in the blood were compared for each animal prechallenge and with blood collected at the time of postmortem using one‐way anova (with some data transformed by log_10_ to ensure normal distribution). The bacterial load between challenge strain was determined using a mixed‐model analysis (*t *= ≤2*,* indicating a significance). The Mann–Whitney *U*‐test was used to compare the severity of the pathological features observed in the organs. Other statistical analysis was performed as described in the relevant section of the text.

## Results

### Dose ranging study – disease presentation

**Figure 1:**
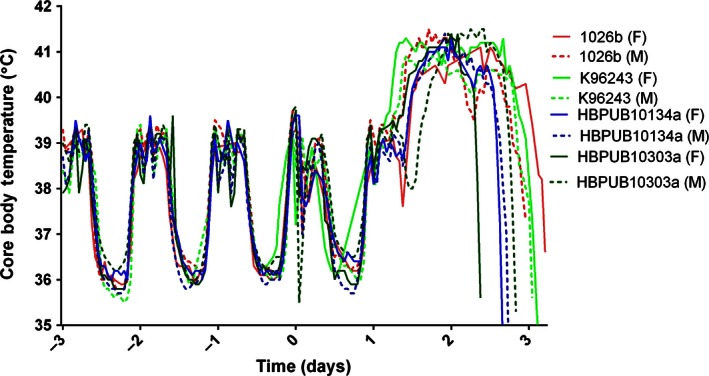
Representative temperature profile for marmosets challenged with *Burkholderia pseudomallei* by the inhalational route. Four strains of *B. pseudomallei* strains (1026b, K96243, HBPUB10303a and HBPUB10134a) were challenged with approximately 1 × 10^2^ cfu, and fever was apparently at approximately 30 h postchallenge. M and F denote male and female respectively. Animals were humanely killed when their core body temperature decreased by 2°C below their normal baseline body temperature for that time of day or night.

Dosing ranging studies were performed for all four strains of *B. pseudomallei* (strains K96243, 1026b, HBPUB10303a and HBPUB10134a). Cohorts of ten animals were challenged for 10 min with target doses of either 10^1^ (‘low’) or 10^2^ (‘high’) cfu of the appropriate *B. pseudomallei* strain (six animals at the ‘high’ dose and four animals at the ‘low’ dose). Group sizes were selected based on Power analysis on a two‐sample *t*‐test of the bacteriology, liver function enzyme and platelet counts (three key read‐outs) on previous data (Nelson *et al*. 2011). Variability was observed in the accumulated volume of the bacteria‐infected air each animal breathed due to the different breathing rates of animals during exposure. This resulted in a variability of doses the animals received; lung‐deposited doses were between 1 and 9 cfu for the low dose and 11 and 202 cfu for the high dose (Table 2). Generally, clinical signs were apparent following the onset of fever (defined as >40°C and loss of the diurnal rhythm). This was typically started 48 h postchallenge (p.c.) for animals that were exposed to the high dose and was similar for all challenge strains (Figure 1). Fever occurred approximately 24 h later for animals exposed with the low challenge dose in a dose‐related fashion as previously described (Nelson *et al*. 2011). An exponential decrease in the time to onset of fever with an increase in dose was apparent (data not shown) (*P* < 0.001). However, there was no difference between strains in the time to onset of fever (*P* = 5.16).

**Table 2:** The survival and challenge dose that marmoset received following inhalational challenge with various strains of *Burkholderia pseudomallei*

Dose (cfu)	Time to humane endpoint (h)	*B. pseudomallei* strain	Notional dose level
1	n/a	HBPUB10134a	Low
1	87.1	1026b	Low
1	108.2	1026b	Low
2	n/a	1026b	Low
2	61.5	HBPUB10303a	Low
2	70.2	HBPUB10134a	Low
3	n/a	HBPUB10303a	Low
3	67.6	HBPUB10303a	Low
3	81.7	K96243	Low
5	n/a	HBPUB10134a	Low
5	100.2	1026b	Low
6	n/a	K96243	Low
6	69.9	HBPUB10303a	Low
6	78.2	K96243	Low
8	75.5	K96243	Low
9	80.4	HBPUB10134a	Low
a10	66.1	HBPUB10303a	High
11	59.6	HBPUB10134a	High
12	53.4	HBPUB10134a	High
22	75.9	1026b	High
24	63.8	HBPUB10134a	High
26	72.9	1026b	High
35	84.0	1026b	High
38	67.4	HBPUB10303a	High
42	75.5	K96243	High
44	59.8	HBPUB10134a	High
47	59.8	HBPUB10303a	High
50	62.6	HBPUB10303a	High
53	58.4	HBPUB10303a	High
55	73.0	K96243	High
72	75.3	K96243	High
81	85.3	1026b	High
95	66.1	K96243	High
97	56.3	HBPUB10303a	High
101	60.3	HBPUB10134a	High
115	69.9	1026b	High
126	61.4	K96243	High
128	84.1	1026b	High
155	61.7	HBPUB10134a	High
202	72.7	K96243	High

a Plethysmography data used to calculate this value should be treated with calculate; it is considered to be associated with a leak in the neck seal that was not detected at the time of challenge.

All animals that became febrile and exhibited clinical signs succumbed to lethal disease with a variation in the time to reach the humane endpoint (Table 2). A strong relationship was observed between the challenge dose the animals received and the time to reach the humane endpoint. Interstrain comparison of this relationship using analysis of covariance (ancova) showed a significance in the virulence of the bacterial strains; *B. pseudomallei* strains HBPUB10303a and HBPUB10134a are more virulent than both strains K96243 (*P* < 0.001 and *P* = 0.03 respectively) and 1026b (both *P* < 0.001), but not from each other (*P* = 0.813). *Burkholderia pseudomallei* strain K96243 is slightly more virulent than strain 1026b (*P* = 0.023). For each strain of *B. pseudomallei*, at least one animal exposed to a low dose remained afebrile (two animals following challenge with *B. pseudomallei* strain HBPUB10134a) and survived until the end of study.

### Characterization of the disease progression

The disease caused by two strains of *B. pseudomallei,* strains K96243 and HBPUB10303a, was further characterized in separate time‐course studies. These were selected due to their virulence, as strain K96243 was the most virulent laboratory strain and strain HBPUB10303a was the most virulent clinical isolate. This was based on the number of animals that succumbed to disease and the time to reach the humane endpoint. Cohorts of 16 marmosets were challenged by the inhalational route with a challenge dose of 48 ± 2 or 31 ± 5 cfu of *B. pseudomallei* strain K96243 or HBPUB103030a respectively. Four animals were humanely killed at each of 4 time points p.c.: 12 h: 24 h, 36 h and 48 h. The time points were based on the previous temperature profile following infection (Figure 1). This represent prefever (12 h), early temperature rise (24 h), late temperature rise (36 h) and fever plateau phase (48 h).

**Figure 2:**
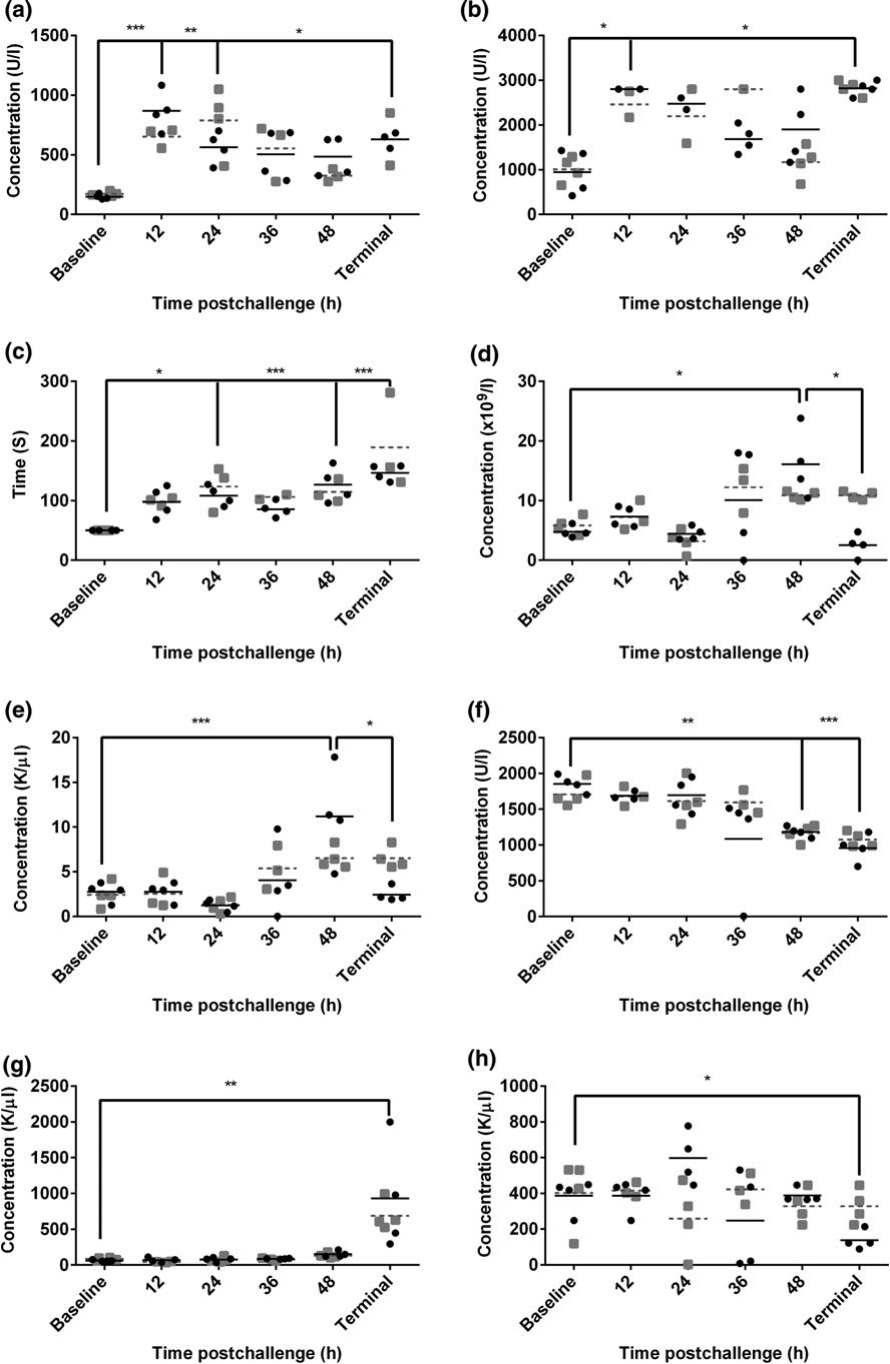
Blood parameters observed in marmosets at the time of killing following inhalational challenge with *Burkholderia pseudomallei*. Data represent individual data points, with data from *B. pseudomallei* strains K96243 and HBPUB10303a being represented by black closed circles and closed grey squares respectively. The mean of each strain is indicated by a black solid line and grey dashed line for strains K96243 and HBPUB10303a respectively. (a) aspartate aminotransferase (AST), (b) lactate dehydrogenase (LDH), (c) activated partial thromboplastin time (aPPT), (d) white blood cell count (WBC), (e) absolute neutrophil count (NEU), (f) amylase (AMYL), (g) alkaline phosphatase (ALKP), (h) platelets (PLT). One‐way anova was performed to determine significant changes in levels, where * is *P* < 0.05, **P = 0.01, ***P = 0.001. No statistical significance was observed between the two strains of *B. pseudomallei*.

At 12 h p.c., all animals had a normal core body temperature and had no overt clinical signs. Bacteria were only recovered from the lungs of 50% of the animals. A significant increase in the levels of aspartate aminotransferase (AST) (*P* < 0.001) and lactate dehydrogenase (LDH) (*P* = 0.01) was observed compared to baseline levels (Figure 2a,b). Histologically, there were no disease‐related pathological features. However, mild interstitial pneumonia was observed in all animals, which is likely to be related to the aerosolization process rather than the disease.

By 24 h p.c., animals had a low‐grade fever and an increase in bacterial burden in the lungs was observed in all but one animal with a median bacterial concentration of 1.5 × 10^5^ cfu in the lungs. Early indications of bacterial dissemination were observed, with <200 cfu/g observed in the spleens and livers. A significant increase in the activated partial thromboplastin clotting time (aPPT) compared to baseline was observed (*P* = 0.01) (Figure 2c). Histologically, some animals exhibited acute bronchopneumonia and alveolitis present.

By 36 h p.c., fever was apparent and there was evidence of further bacterial dissemination in all organs except the kidney and brains. However, only low levels of bacteraemia were detected at <100 cfu/ml. Haematological analysis at this time shows evidence of a non‐significant increase in levels of white blood cells (WBC) and a significant increase in the NEU level compared to baseline (*P* < 0.001) (Figure 2d,e). Histologically, there were further pathological features in the lungs of animals, with the presence of more extensive acute bronchopneumonia and mild to moderate interstitial pneumonia.

By 48 h p.c., mild clinical signs were apparent and a high fever was observed. Bacterial burden increased in infected organs, and bacteria were consistently present in all other organ types with a low level of bacteraemia. There was a significant increase in the levels of globulin (GLOB; *P* = 0.01; data not shown) and WBC (*P* = 0.01 and NEU (*P* = 0.01) (Figure 2d,e), as well as a significant decrease in the levels of amylase (AMYL) (*P* > 0.001) compared to baseline levels (Figure 2f). Histologically, there were increased foci of necrosis in all tissues.

‘Terminal’ animals (those that had reached the humane endpoint i.e. a decrease in temperature by 2°C compared to the baseline temperature) exhibited more pronounced clinical signs, increased bacterial burden and bacteraemia. Additional liver dysfunction was evident with significantly increased levels of AST compared to baseline (*P* < 0.05) and with significantly increased levels of alkaline phosphatase (ALKP; *P* = 0.01; Figure 2g) and gamma‐glutamyl transferase (GGT; *P* < 0.05; data not shown) observed for the first time. A significant decrease in the levels of AMYL (*P* = 0.001) and platelets (PLT; *P* < 0.05) was observed (Figure 2f,h). At this time, the more extensive and severe pathological features were observed in the lung, liver and spleen, with acute to necrotizing bronchopneumonia and multifocal acute hepatitis and splenitis.

In general, the pathological features observed between the two strains of *B. pseudomallei* were very similar, and no statistically significant differences were observed in the severity of progression of the disease using a Mann–Whitney comparison.

### Histology

**Figure 3:**
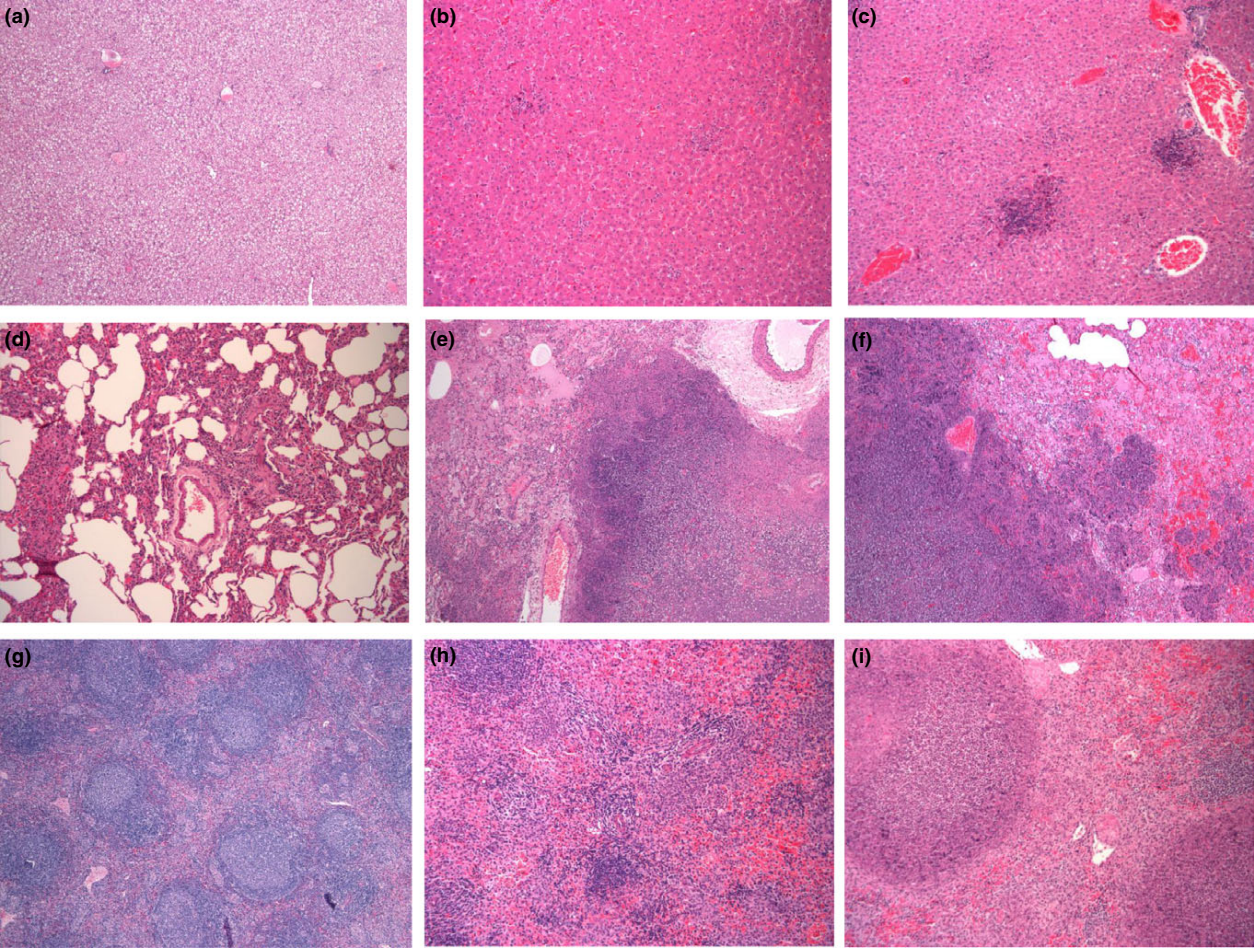
Representative H&E tissue sections from marmosets following inhalational challenge with *Burkholderia pseudomallei*. Liver (a) euthanised 12 h post‐challenge with strain HBPUB10303a, 5 × 1, (b) and (c) succumbed to disease following challenge with strain 1026b (severity score 1, 10 × 1) and strain HBPUB10303a (severity score 4, 10 × 1), respectively. Lung (d) euthanised 12 h post‐challenge with strain HBPUB10303a, 10 ×1, (e) and (f) succumbed to disease following challenge with strain 1026b (severity score 4, 5 × 1) and strain HBPUB10303a (severity score 4, 5 × 1), respectively. Spleen (g) euthanised 12 h post‐challenge with strain HBPUB10303a, 5 × 1, (h) and (i) succumbed to disease following challenge with strain 1026b (severity score 1, 10 × 1) and strain HBPUB10303a (severity score 4, 10 × 1), respectively.

**Figure 4:**
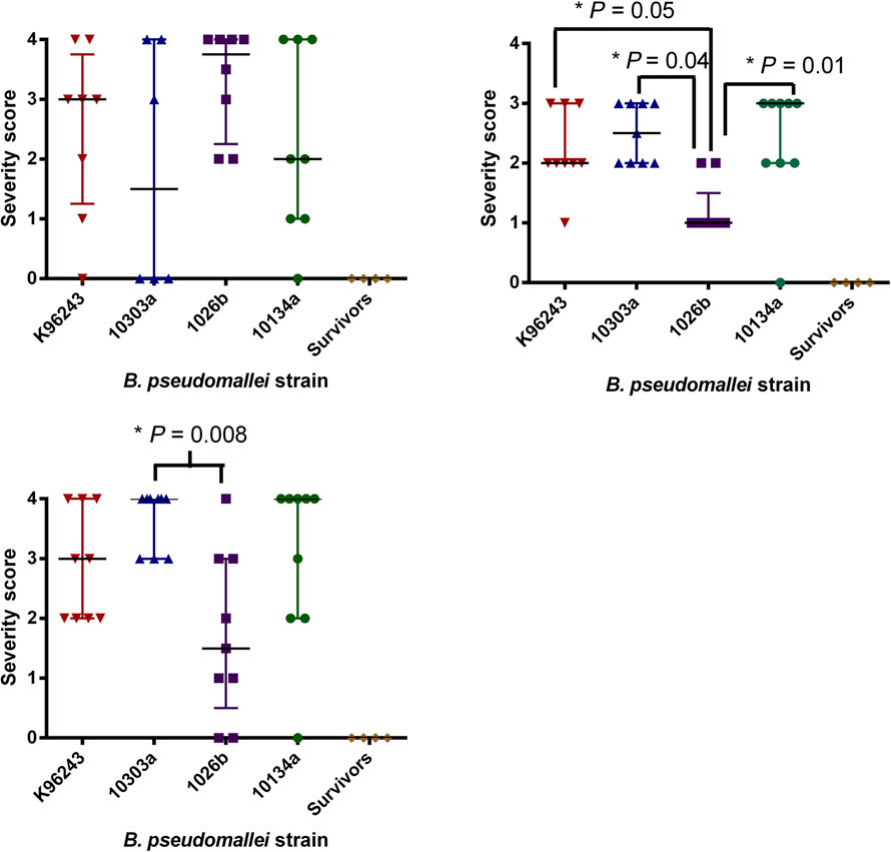
Comparison of the severity scores in various marmoset organs at the humane endpoint following inhalational challenge with one of the four strains *of Burkholderia pseudomallei*. (a) Lung, (b) Liver (c) Spleen.

Following exposure to *B. pseudomallei*, multifocal necrotizing hepatitis and splenitis were observed in all animals that succumbed to disease regardless of the challenge strain. The severity of the lesions in terminal animals was determined, and there was no difference in the severity of lesions in relationship to the challenge dose the animals received. However, the severity of the lesions caused by *B. pseudomallei* strain 1026b differed from those caused by the other strains (Figure 3). Animals challenged with *B. pseudomallei* strain 1026b had significantly less severity and extension of lesions in liver (*P* < 0.05) and spleen (*P* = 0.008 for strain HBPUB10303a only) (Figure 4). Typically, the severity of lesions in the liver and spleens of animals challenged with *B. pseudomallei* strain 1026b was scored at <2, whereas following challenge with the other strains of *B. pseudomallei,* the severity of lesions was typically scored at 3 or 4, but occasionally 2. There was no correlation between the severity score in these organs and the bacterial burden in the organs (*R* ^2^ = 0.187, *P* = 0.28 and *R* ^2^ = 0.425, *P* = 0.08, for the spleen and liver of animals challenged with strain 1026b).

Severe multifocal necrotizing pneumonia was evident in all animals challenged with the high dose of *B. pseudomallei*, regardless of the infecting strain, with severity scores in terminal animals of typically 4 and occasionally 2 or 3. However, the identification of lesions in animals challenged with low doses of *B. pseudomallei* was sporadic. This may be related to the fewer bacteria introduced into the lungs at challenge which establish infection at fewer sites before the systemic spread of disease, resulting in a non‐uniform distribution of lesions which occasionally were not recovered for histological analysis. Following challenge with *B. pseudomallei* strain 1026b, the frequency of lesions was partially sporadic although the severity of lesions was generally greater, with typical scores of 3 or 4. There were no pathological features observed in the brains or pancreas of any animal, and the pathological features observed in the kidney were considered to be background pathology.

In all cases, the five animals that survived challenge (one animal each challenged with strains HBPUB10303a, 1026b and K96243 and two animals that were challenged with HBPUB10134a) had no significant changes observed in liver, spleen and lungs.

No correlation was observed between the challenge dose the animals received and the pathological features (Spearman's correlation analysis) or time to death (*P* < 0.001).

### Lesion progression and cellular composition

Immunohistochemistry was performed on selected tissues (liver, spleen and lungs) from animals challenged with *B. pseudomallei* strain K96243 and HBPUB10303a, as the more virulent laboratory strain and clinical isolate respectively (Tables 3 and 4). IHC was performed to allow the location of bacteria using bacterial capsular antigen, 3VIE5 (Jones *et al*. 2002). Characterization of cellular populations within the lesions was also performed assessing macrophages and NEUs (MAC387^+^), T cells (CD3^+^), B cells (CD79^+^) and iNOS^+^ cells.

**Table 3:** Lesion histopathology and characterization in various organs of marmosets challenged with *Burkholderia pseudomallei* strain K96243 by the inhalational route

Time (h)	Animal ID	Lung						Spleen						Liver					
		H	Bp	M	T	B	i	H	Bp	M	T	B	i	H	Bp	M	T	B	i
12	125R	0	0	0	0	0	0	0	0	0	0	0	0	0	0	0	0	0	0
	95P	0	0	0	0	0	0	0	0	0	0	0	0	0	0	0	0	0	0
	136R	0	0	0	0	0	0	0	ND	ND	ND	ND	ND	0	0	0	0	0	0
	66N	0	0	0	0	0	0	0	0	0	0	0	0	0	0	0	0	0	0
24	51R	0	0	0	0	0	0	0	0	0	0	0	0	0	0	0	0	0	0
	76P	0	0	0	0	0	0	0	0	0	0	0	0	0	0	0	0	0	0
	9R	0	1	0	0	2	1	0	0	0	0	0	0	0	0	0	0	0	0
	114R	1	0	0	0	0	0	ND	ND	ND	ND	ND	ND	0	0	0	0	0	0
36	94P	0	0	0	0	0	0	0	0	0	0	0	0	0	0	0	0	0	0
	124R	2	3	1	1	4	2	2	1	1	1	4	2	1	0	0	0	3	2
	54P	1	0	ND	0	0	0	0	0	ND	0	0	0	0	0	ND	0	0	0
	19R	2	3	2	1	4	2	1	1	0	0	0	0	1	0	0	1	3	2
48	208M	1	1	1	0	3	1	1	2	0	0	4	2	1	1	0	0	2	2
	48R	2	4	0	0	4	2	2	2	0	0	4	2	1	1	0	0	3	2
	107R	0	0	0	0	0	0	0	1	0	0	0	0	1	1	0	1	3	2
	140R	2	4	1	1	4	2	1	2	0	1	4	2	1	1	1	0	3	2
Terminal	85P	2	4	1	0	4	2	2	4	1	1	4	0	2	3	0	0	3	0
	66P	4	4	1	ND	4	2	2	2	0	ND	4	2	1	1	0	ND	2	2
	64N	3	4	2	1	4	2	3	4	1	1	4	1	2	3	1	0	3	1
	15R	3	4	0	0	4	1	3	4	0	0	4	1	3	3	0	0	3	1

H = average score of lesion severity; Bp = average score of the presence of bacteria in the lesion detected by IHC; M = average score of the presence of macrophages in the lesion detected by IHC; T = average score of the presence of T cells in the lesion detected by IHC; B = average score of the presence of B cells in the lesion detected by IHC; i = average score of the presence of nitric oxide synthase in the lesion detected by IHC; ND, not determined; IHC, immunohistochemistry.

**Table 4:** Lesion histopathology and characterization in various organs of marmosets challenged with *Burkholderia pseudomallei* strain HBPUB10303a by the inhalational route

Time (h)	Animal ID	Lung						Spleen						Liver					
		H	Bp	M	T	B	i	H	Bp	M	T	B	i	H	Bp	M	T	B	i
12	9T	0	0	0	0	0	0	0	0	0	0	0	0	0	0	0	0	0	0
	139R	3	0	0	0	0	0	2	0	0	0	0	0	2	0	0	0	0	0
	119R	0	0	0	0	0	0	0	0	0	0	0	0	0	0	0	0	0	0
	133P	0	0	0	0	0	0	0	0	0	0	0	0	0	0	0	0	0	0
24	8T	1	1	2	1	3	2	0	0	0	0	0	0	0	0	0	0	0	0
	105R	0	0	0	0	0	0	0	0	0	0	0	0	0	0	0	0	0	0
	2T	1	1	0	0	2	2	0	0	0	0	0	0	0	0	0	0	0	0
	69R	0	0	0	0	0	0	0	0	0	0	0	0	0	0	0	0	0	0
36	19T	0	0	0	0	0	0	0	2	0	0	0	2	0	1	0	0	1	0
	14R	2	4	1	1	4	2	0	0	0	0	0	0	0	0	0	0	0	0
	128R	2	3	3	1	4	2	0	0	0	0	0	0	0	0	0	0	0	0
	109R	0	0	0	0	0	0	1	1	0	0	3	0	1	1	0	0	3	1
48	132R	2	3	1	1	4	2	2	2	0	0	4	2	0	0	0	0	3	2
	76R	0	0	0	0	0	0	2	3	0	1	4	2	2	0	1	1	3	2
	154R	0	4	1	1	4	2	0	3	0	0	4	2	0	2	0	0	4	2
	18R	2	3	1	1	4	2	1	2	1	1	4	2	1	1	1	1	4	2
Terminal	222N	4	4	1	1	4	2	3	4	0	1	4	1	2	3	0	0	3	1
	62P	0	2	1	0	2	1	4	4	0	0	4	1	2.5	3	0	1	3	1
	23P	3	4	1	0	4	1	4	4	0	0	4	0	3	3	0	0	3	1
	111R	1	2	1	0	2	2	4	4	0	1	4	2	2	3	0	0	3	0

H = average score of lesion severity; Bp = average score of the presence of bacteria in the lesion detected by IHC; M = average score of the presence of macrophages in the lesion detected by IHC; T = average score of the presence of T cells in the lesion detected by IHC; B = average score of the presence of B cells in the lesion detected by IHC; i = average score of the presence of nitric oxide synthase in the lesion detected by IHC; ND, not determined; IHC, immunohistochemistry.

At 12 h following challenge with *B. pseudomallei*, there were no detectable lesions or bacterial antigen in any of the tissues examined. At 24 h p.c., small histological lesions were occasionally observed in the lungs only. These lesions were associated with small to moderate numbers of macrophages and NEUs and a moderate number of T cells. The macrophages and NEUs were observed particularly in the alveoli and adhering to the blood vessel endothelium, and these cells had a moderate level of iNOS expression. T cells were also observed in the periphery and in nearby capillaries. Bacteria were associated predominately with the macrophages within the lesion and occasionally with NEUs. Bacteria were also observed in two small areas lining the surface of alveolar cells and terminal bronchioles in one animal. This was associated with the presence of a small number of macrophages and a slight increase in the number of T cells.

At 36 h p.c., moderate to abundant numbers of bacteria within macrophages and NEUs were observed in the centre of the pulmonary lesions. The lesions primarily consisted of macrophages and NEUs infiltrating the alveoli and also located in the peribronchial and perivascular areas resulting in a thickening of the alveolar septa. Numerous iNOS‐expressing macrophages were observed within the lesions and also in distal areas. There was an increase in the number of T cells associated with the lesions and in neighbouring areas. Only a few scattered B cells were observed. Macrophages and NEUs were the most abundant cell type observed in the few mild histological lesions in the liver and spleens, although T cells were also observed. Some of the macrophages expressed iNOS.

**Figure 5:**
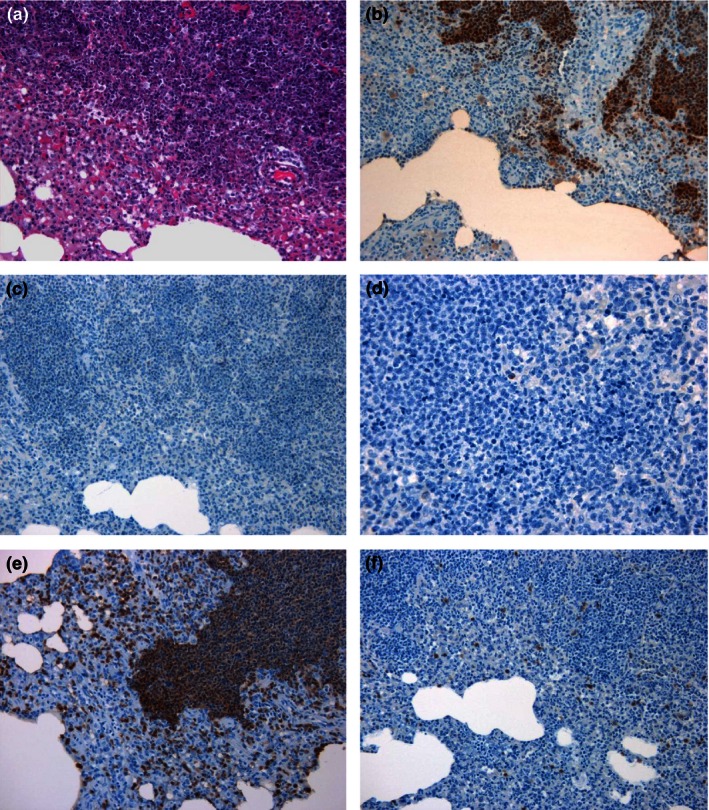
Representative lung sections from marmosets 48 h following inhalational challenge with *Burkholderia pseudomallei* HBPUB10303a. (a) Histological lesion (20×), (b) *B. pseudomallei* antigen stained brown (20×), (c) nitric oxide synthase expressing cell marker stained brown (20×), (d) B‐cell marker stained brown (20×), (e) macrophage/neutrophil marker stained brown (20×), (f) T‐cell marker stained brown (20×).

**Figure 6:**
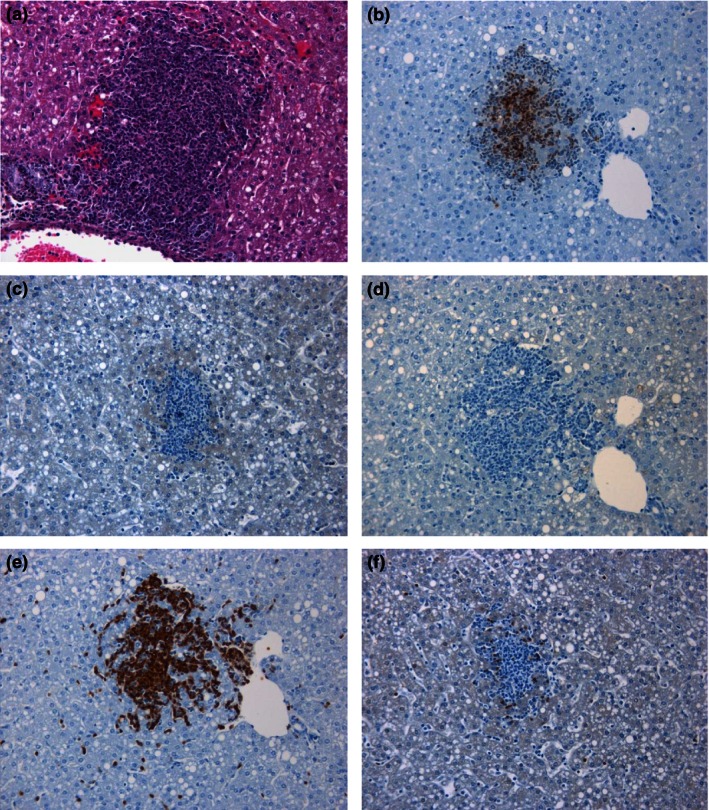
Representative liver sections from marmosets 48 h following inhalational challenge with *Burkholderia pseudomallei* HBPUB10303a. (a) Histological lesion (20×), (b) *B. pseudomallei* antigen stained brown (20×), (c) nitric oxide synthase expressing cell marker stained brown (20×), (d) B‐cell marker stained brown (20×), (e) macrophage/neutrophil marker stained brown (20×), (f) T‐cell marker stained brown (20×).

**Figure 7:**
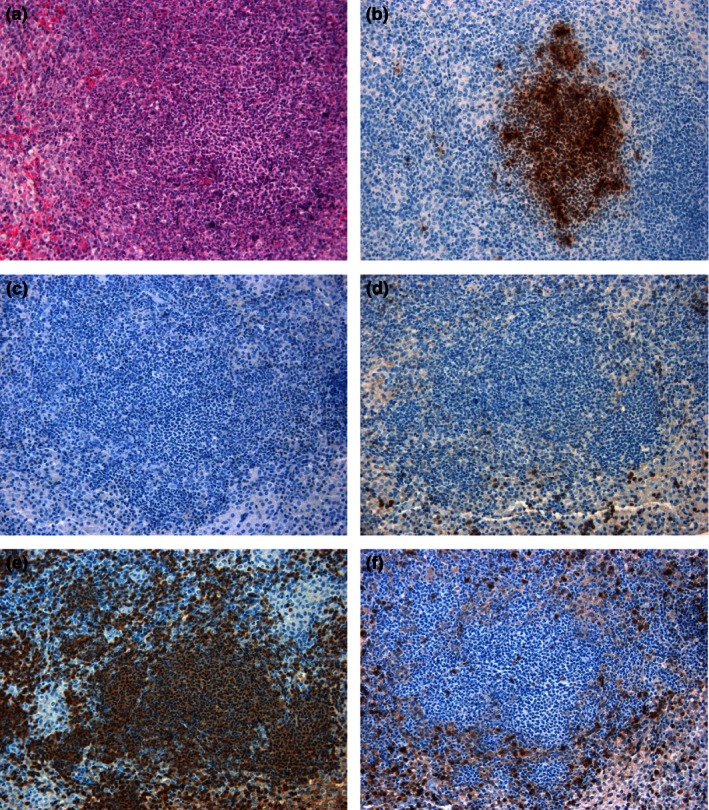
Representative spleen sections from marmosets 48 h following inhalational challenge with *Burkholderia pseudomallei* HBPUB10303a. (a) Histological lesion (20×), (b) *B. pseudomallei* antigen stained brown (20×), (c) nitric oxide synthase expressing cell marker stained brown (20×), (d) B‐cell marker stained brown (20×), (e) macrophage/neutrophil stained brown, (f) T‐cell marker stained brown (20×).

At 48 h p.c., moderate to abundant numbers of macrophages/NEUs were observed both within pulmonary lesions and in the surrounding tissues (Figure 5). However, the number of macrophages expressing iNOS and the number of T cells did not appear to increase substantially. Bacteria were frequently observed with the macrophages and NEUs, both within the centre of lesions and in the bronchiolar lumina, peribronchial and perivascular spaces. An increase in the number of macrophages and NEUs was observed in the sinusoids of the liver, and bacteria were associated with infiltrating macrophages and Kupffer cells (Figure 6). In the spleen, immunolabelled bacteria were located primarily within macrophages in the centre of small lesions, surrounded by NEUs not containing bacteria. In larger lesions, the NEUs were also associated with the presence of bacteria (Figure 7). Similar to the lungs, only a small number of T cells were observed.

There was no statistically significant difference in the cellular composition of pulmonary lesions with time (Kruskal–Wallis comparison). However, the amount of macrophages, T cells and bacterial antigen changed with time in the liver (*P* < 0.017). The levels of bacterial antigen associated with the lesions increased with time in the spleen (*P* = 0.016).

## Discussion

Marmosets are susceptible to *B. pseudomallei* strain 13392 by both the inhalational and subcutaneous routes of infection resulting in acute lethal disease (Nelson *et al*. 2011, 2014). Following inhalational challenge with either *B. pseudomallei* strain K96243 or HBPUB10303a, there is an initial, rapid proliferation of the bacteria in the lungs of the animals. Bacterial numbers increased from approximately 40 cfu to 1.5 × 10^5^ cfu within 24 h, indicating a doubling time of approximately 2 h. Reports in the literature suggest that the doubling time of *B. pseudomallei* ranges from 1.5 to 2.3 h *in vitro* and approximately 6 h in the human macrophage‐like U937 cell line (Lee *et al*. 2007; Chieng *et al*. 2012). This appears to be highly dependent on the bacterial strain and indeed the isolate. Bacterial proliferation in the lung of marmosets appears to occur at a rate comparable to the *in vitro* doubling time of these strains performed in‐house (data not shown). This indicates a very limited innate control of the bacterial replication in the marmoset lung; however, the doubling rate is consistent with the replication in the lungs of Balb/c mice challenged with a similar dose (Lever *et al*. 2009). While the marmoset is highly susceptible to *B. pseudomallei*, it still has utility in assessing subtle differences between strains. Previously, an acute model of melioidosis, the hamster, was utilized as a tool to assess the virulence of type III secretion deletion mutants (Warawa & Woods 2005). The marmoset model identified statistically significant increases in virulence of the two recent clinical isolates tested (strains HBPUB10303a and HBPUB10134a) compared to the two laboratory strains (strains K96243 and 1026b). This increased virulence of *B. pseudomallei* strain HBPUB10303a compared to strain K96243 was reported to be evident in the murine model with LD_50_'s of 1 and 4 cfu respectively (Massey *et al*. 2014). However, in that study, no statistical significance in the result was stated. Therefore, the approach used in the marmoset model appears to be a better way of detecting these subtle differences.

Despite the difference in virulence, the data presented in the current study indicate that the disease progression in the marmoset and clinical readouts were similar for a further four strains of *B. pseudomallei*, with some interstrain differences in pathological features and survival observed. This suggests that the strain of *B. pseudomallei* is not the most important factor affecting the disease presentation of melioidosis. Lethal melioidosis in the marmoset, regardless of the infecting strain, presented as acute, febrile disease exhibiting many manifestations of human melioidosis including characteristic pathological features, coagulopathy and liver dysfunction (Dance 1996; Mukhopadhyay *et al*. 2004; Wiersinga *et al*. 2008). Other factors, such as the inoculating dose, host predisposition, genetic factors, must have a more important role in the disease presentation than the strain. Indeed, in the study presented here, the challenge dose the animal received had a more marked effect on the disease outcome in two ways. Firstly, the higher the challenge dose, the shorter the survival. Secondly, lethality was observed consistently following challenge with >10 cfu of *B. pseudomallei*. However, at challenge doses of <6 cfu, there was at least one animal per infecting strain that survived to the end of the study.

Similar to the disease presentation, the histopathological analysis of marmoset tissue was generally consistent between the infecting strains of *B. pseudomallei*. Necrotizing hepatitis, splenitis and pneumonia were observed that is consistent with human melioidosis (Piggot & Hochholzwe 1970; Wong 1995). However, the severity and extension of the histopathological changes observed in the liver, spleen and lungs of animals challenged with *B. pseudomallei* strain 1026b was distinctly different. The pathological features were less severe in the reticuloendothelial organs; however, they were more severe, if less frequent, in the lungs. This was especially evident in two marmosets that received <6 cfu of *B. pseudomallei* strain 1026b, which had a protracted disease. This may be a feature of the low challenge dose these animals received. However, this was also apparent in higher challenge dose animals and may indicate a greater capability by this strain of *B. pseudomallei* to colonize pulmonary tissue. This was also associated with a delayed systemic infection and may also be related to this bacterial strain being isolated from a non‐lethal human case. All other strains used in this study were from lethal, pneumonic cases of melioidosis. Therefore, the ability to successfully form lesions in the lung may be a method of controlling bacterial dissemination and increasing survival rate.

This study also provided evidence on how the characteristic melioidosis lesions are formed. Bacteria initially infected resident macrophages following phagocytosis in all organs. This is followed by an initial infiltration of the surrounding area by NEUs, most likely attracted by chemical mediators, of which iNOS may play a role. As the influx of inflammatory cells progresses, the number of macrophages and NEUs within the lesion increases and other cells are also present, including T lymphocytes and rarely B cells. This results in a progressive increase in lesion size and extension of the inflammatory changes to the surrounding tissue. This includes increased numbers of leucocytes in the neighbouring vascular structures and increased attachment of leucocytes to endothelium and diapedesis. After the infiltration of inflammatory cells, the bacteria will induce the necrosis of both the leucocytes and tissue parenchyma.

In conclusion, the marmoset model was used to determine that the infecting dose had a greater effect on the temporal response to *B. pseudomallei* than the infecting strain. The model was also able to distinguish a subtle difference in the severity of lesions following infection with strain 1026b; however, all lesions were formed following macrophage phagocytosis and grew in size following the recruitment of NEUs to the site.
